# Socioeconomic and demographic factors determining the underweight prevalence among children under-five in Punjab

**DOI:** 10.1186/s12889-020-09675-5

**Published:** 2020-11-30

**Authors:** Rizwan Farooq, Hina Khan, Masood Amjad Khan, Muhammad Aslam

**Affiliations:** 1 Bureau of Statistics, Government of the Punjab Lahore, Lahore, Pakistan; 2 Department of Statistics, GC University Lahore, Lahore, Pakistan; 3 Department of Statistics, Faculty of Science, King Abdulaziz University, Jeddah, 21551 Saudi Arabia

**Keywords:** Bayes X, Fully Bayesian approach, Geo-additive models, Locality, Markov chain Monte Carlo, Stunting, Underweight, Wealth index quintile, Wasting

## Abstract

**Background:**

Underweight prevalence continues to be major public health challenge worldwide, particularly in developing countries like Pakistan. This study is focused on socio-economic and demographic aspects of underweight prevalence among children under-five in Punjab.

**Methods:**

In this study, several socioeconomic and demographic factors are considered using MICS-4 data-set. Only those variables which are usually described in the nutritional studies of children were picked. Covariates include: the age of children, sex of the children, age of mother, total number of children born to women, family wealth index quintile, source of drinking water, type of sanitation, place of residence, parents’ education and occupation. All Categorical variables are effect coded. The child’s age and the mother’s age are assumed to be nonlinear, geographical region is spatial effect, while other variables are parametric in nature.

Since, the response is binary, covariate comprises linear terms, nonlinear effects of continues covariates and geographic effects, so we have use Geo-additive models (based on Fully Bayesian approach) with binomial family under logit link. Statistical analysis is performed on Statistical package R using Bayes X and R2 Bayes X Libraries.

**Results:**

Underweight status of children was found to be positively associated with number of under-five children in household, total number of children ever born to women and age of mother when the child was born. Whereas, it negatively associated with place of residence, parent’s education and family wealth index quintile. On the regional effect, the Southern Punjab has higher prevalence of underweight compared to Central and Northern Punjab.

**Conclusion:**

Similarity of our results with several other studies demonstrate that the Geo-additive models are an ideal substitute of other statistical models to analyze the underweight prevalence among children. Moreover, our findings suggest the Punjab Government, to introduce target-oriented programs such as poverty reduction and enhancement of education and health facilities for poor population and disadvantaged regions, especially Southern Punjab.

**Supplementary Information:**

**Supplementary information** accompanies this paper at 10.1186/s12889-020-09675-5.

## Background

Underweight prevalence is a major cause of child mortality worldwide. Underweight children have lesser immunity against infections and have higher chance to die from common diseases, and those who survive are exposed to recurring illnesses and slower growth. Such children are more likely to have lower IQ, which not only affect their educational performance but also reduce their working abilities [1, 2].

**Fig. 1:**
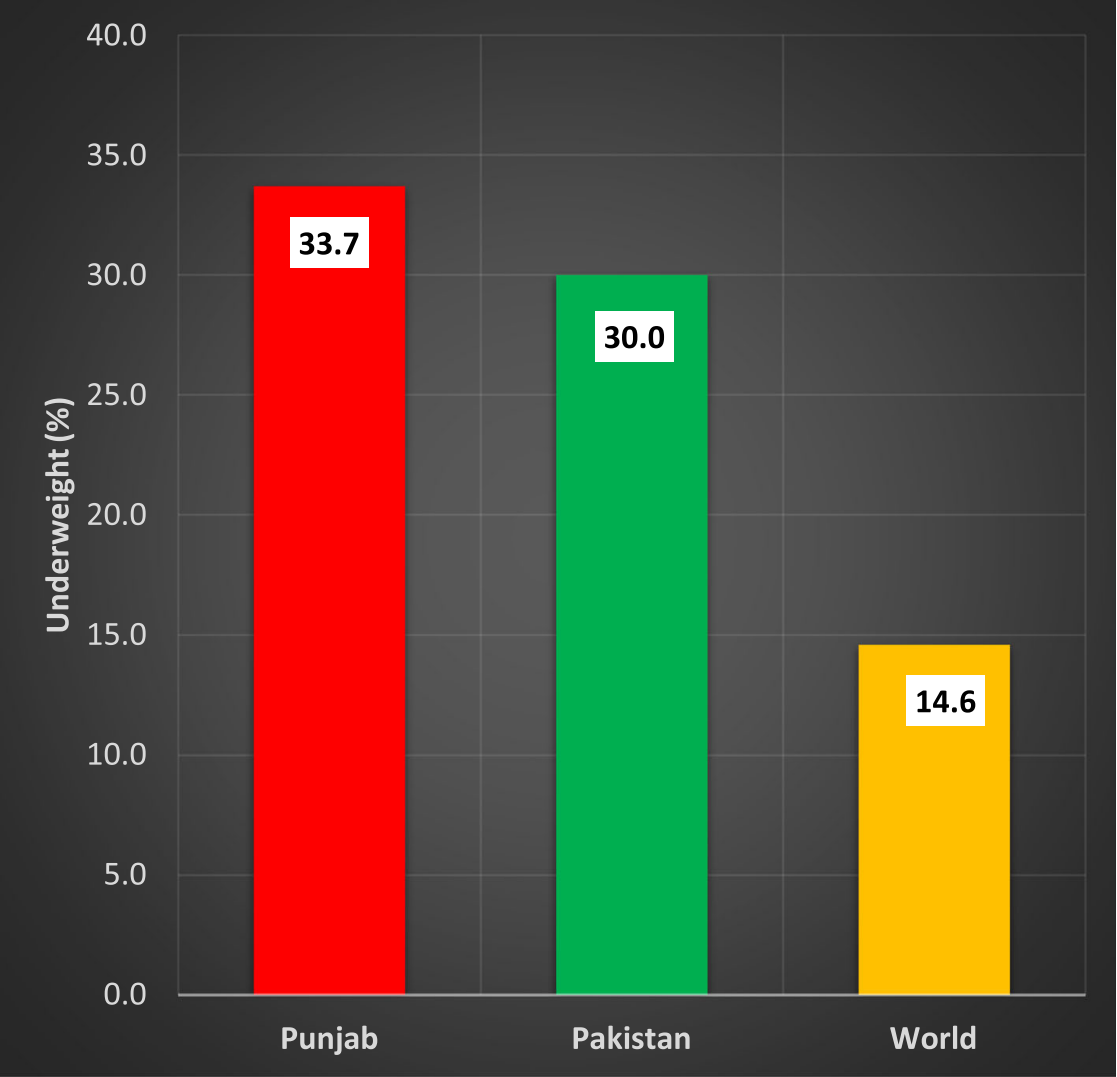
Comparison of underweighted children under-five in Punjab, with Pakistan and World 2014. A self-explanatory bar chart created through Microsoft excel.The figures pertaining to “Punjab”, “Pakistan” and “World” are taken from MICS-14, PDHS (2012–13) and the Website of world bank respectively [1, 3, 4].

According to MICS-2014 [1], the prevalence of underweight among under-five children in Punjab is 33.7%, which is higher than the overall the underweight prevalence in the country (Fig. 1). It is a point of serious concern, because Punjab is comparatively most developed province of Pakistan regarding health and educational facilities. Additionally, it is also the most densely populated province of country, so a minor change in the percentage can be of greater significance. Therefore, a question arises what are the factors behind such a high rate of underweight prevalence among children in the province.

To the best of our knowledge, not even a single research study has been conducted in Punjab so far, to explore and analyze the impact of factors influencing such a high proportion of underweight.

Alasfoor et al. (2007), Mengistu et al. (2013) and Rayhan et al. (2006), use bivariate and multivariate analysis to identify the causes of under-five malnutrition [5–7]. The key factors for under-five malnourishment were the education level of parents, income of the household and total number of under-five children in household. Sapkota & Gurung (2009) and Wolde et al. (2014) used logistic regression techniques to calculate the underweight prevalence among children under-five [8–10]. They discovered that the socioeconomic status of family, maternal education and gender, ethnicity and age of children were closely linked with underweight prevalence. Some other authors [11–16] found that age and gender of children, child’s malaria status, exclusive breastfeeding, child’s vaccination status, maternal education, parent’s access to media, ARI in children, poverty and the type of toilet used in household were strongly correlated with underweight.

Majority of previous studies on child’s underweight have usually focused on different socio-economic, demographic or health related factors but mostly have ignored spatial and nonlinear effects of covariates. Considering these aspects, Kandala et al. (2008), Lasisi et al. (2014) and Lasisi et al. (2014) use Bayesian geo-additive models to observe the pattern of underweight among the children under five year of age [14, 17, 18]. Their studies showed that underweight among children depends on residence, age of the child, maternal education, wealth status of the household and geographical zone.

## Methods

We aim to study the impact of several socio-economic and demographic factors on underweight status of under-five children in Punjab, considering the spatial and nonlinear effects of covariates. This study is based on data-set of the MICS-2014 conducted by Punjab Bureau of Statistics with the collaboration of UNICEF.

Two-stage cluster sampling technique was used for selection of sample. At first stage 2050 clusters were randomly selected (called primary sampling units) from all 36 districts of Punjab through proportional allocation. On second stage systematic sample of 20 households (Called secondary sampling units) was randomly drawn from each of selected cluster [1]. The information about one or more explanatory variable(s) used in this study was missing for 6789 out of 31,083 children interviewed, so these were discarded. Finally, the total sample size for the research is 10,774 women of reproductive age (15–49) in Gilgit Baltistan.

Underweight status of children (*UWa*), i.e. ‟weight-for-age” is taken as a dependent variable. In this regard, the weight and the height of each child in sample is calculated separately. These measures are then expressed as Z-scores from the median of the reference population.

$$ {\mathrm{Z}}_{\mathrm{i}}=\frac{{\mathrm{WAP}}_{\mathrm{i}-}{\upmu}_{\mathrm{WAP}}}{\upsigma_{\mathrm{WAP}}} $$

Where, WAP_i_ is the weight for age percentile of *i*_*th*_ child, while **μ**_**WAP**_ and **σ**_**WAP**_ denote the median and standard deviation of *WAP* of reference population respectively. A child ‟*i*” is declared as an underweight if *Z*_*i*_ <  − 2, else Normal weight. So, our response variable (*UWa*) is binary with 0 & 1 refers to Normal weight and underweight children respectively.

We consider several socio-economic and demographic covariates as predictor, which are generally described in the nutritional studies of children. Covariates include; the age of children, sex of the children, age of mother, total number of children ever born to women, family wealth index quintile, source of drinking water, type of sanitation, place of residence (locality), parents’ education and occupation. All categorical variables are effect coded; Children’s age and Mother’s age (when child born) are assumed nonlinear; ‟Region” is geographic effect, while other covariates are parametric in nature (Additional file 1).

## Statistical analysis

Since, our response is binary; covariates consist of usual linear terms, nonlinear effects of continuous covariates and geographic effects, such sort of models are called Geo–Additive Regression Models, which are special case of Structure Additive Regression Models. We use Geo–Additive Regression under logit link, because we are interested to find the relative risk of underweight at different levels of given covariates. For inference, we have used a Fully Bayesian Approach because we want to describe them probabilistically. In this approach, all unknown regression coefficients and the smoothing functions are considered as random variables and must be assigned with appropriate prior distributions (Additional file 2). This methodology is based on Markov priors and uses Markov Chain Monte Carlo techniques to draw samples from posterior, and for model checking it normally used the Deviance Information Criterion. For detail, see [19–22].

Statistical analysis is performed on R Language using BayesX and R2BayesX Libraries (Additional file 2).

## Results

Before executing the final model, a bivariate analysis was performed, in order to decide which variables were to be included in the final model. The association between underweight status of children and different socio-economic and demographic variables (based on Pearson Chi-square tests of independence at 5% level of significance) is given in Table 1. According to Table 1, All variables, except the gender of children, found significantly associated with the underweight status of children. It is, therefore, the gender of children is discarded from final model (Additional file 2).

**Table 1:** Association between underweight and socioeconomic and demographic variables

Covariate	Categories	Underweight		Pearson Chi- Square Test		
		No (%)	Yes (%)	Value	d.f	***P***-Value
Locality	Rural	10,095 (62.90)	5954 (37.10)	189.35	1	0.000
	Urban	5915 (71.74)	2330 (28.26)			
Gender	Female	8153 (65.74)	4248 (34.26)	0.276	1	0.607
	Male	7857 (66.06)	4036 (33.94)			
Mother’s education level	None	6880 (43.68)	4998 (56.32)	931.495	4	0.000
	Primary	2975 (66.08)	1527 (33.92)			
	Middle	1695 (71.88)	663 (28.12)			
	Secondary	2209 (76.12)	693 (23.88)			
	Higher	2251 (84.82)	403 (15.18)			
Father’s education level	None	3848 (55.39)	3099 (44.61)	810.205	4	0.000
	Primary	2772 (61.72)	1719 (38.28)			
	Middle	2729 (67.27)	1328 (32.73)			
	Secondary	3877 (51.98)	1456 (27.30)			
	Higher	2784 (80.01)	682 (19.99)			
Mother’s occupation	Housewife	14,345 (66.27)	7300 (33.73)	12.285	1	0.000
	Working Woman	1665 (62.85)	984 (37.15)			
Father’s occupation	Unemployed	836 (68.36)	387 (31.64)	324.201	4	0.000
	Laborer	3606 (13.33)	2667 (42.52)			
	Farmer	2488 (92.58)	1407 (36.12)			
	Official	4800 (36,24)	2065 (30.08)			
	Businessman	4280 (79.50)	1758 (29.12)			
Wealth index quantile	Lowest	2981 (52.44)	2704 (47.56)	1076.13	4	0.000
	Second	3137 (61.11)	1996 (38.89)			
	Middle	3374 (67.64)	1614 (32.36)			
	Fourth	3484 (72.09)	1349 (27.91)			
	Highest	3034 (83.01)	621 (16.99)			
Total number of under-five children in household	None	16,010 (65.90)	8284 (34.10)	22.077	8	0.040
Total children ever born to a woman	None	15,997 (65.90)	8276 (34.10)	192.48	17	0.000
Sanitation	Unimproved	4723 (57.08)	3551 (42.92)	434.23	1	0.000
	Improved	11,287 (70.46)	4733 (29.54)			
Sources of drinking water	Unimproved	850 (62.32)	514 (37.68)	7.1	1	0.008
	Improved	15,098 (65.84)	7832 (34.16)			
Access to media	No	5030 (58.08)	3630 (41.92)	366.011	1	0.000
	Yes	10,980 (70.23)	4654 (29.77)			
Region	Southern Punjab	5029 (60.10)	3339 (39.90)	264.389	2	0.000
	Central Punjab	9491 (67.76)	4516 (32.24)			
	Northern Punjab	1489 (77.63)	429 (23.37)			
Age of mother when children born	None	16,010 (65.90)	8284 (34.10)	54.538	38	0.0400
Age of children	None	16,010 (65.90)	8284 (34.10)	140.544	59	0.000

Model summary for our final model (with 20,000 iterations, burnin = 0, step = 10, family = binomial, link = logit) is given in Table 2 and Table 3 (Additional file 2) (Fig. B1) (Fig. B2).

**Table 2:** Parametric coefficients

Co-efficient		Mean(γ)	Sd	2.50%	50%	97.50%
(Intercept)		−0.2324	0.1096	−0.4543	−0.2308	− 0.0123
Locality	Urban	−0.1254	0.039	−0.0478	− 0.1256	− 0.2026
Mother’s education level	Primary	− 0.0814	0.0403	− 0.1654	− 0.0809	− 0.0042
	Middle	− 0.1904	0.0543	− 0.2979	− 0.19	− 0.0851
	Secondary Secondary	− 0.2502	0.0569	−0.3635	− 0.2505	− 0.139
	Higher	−0.5868	0.0722	−0.7318	− 0.5849	− 0.446
Father’s education level	Primary	−0.0892	0.0419	−0.1725	− 0.0898	− 0.0045
	Middle	−0.173	0.0444	−0.2571	− 0.1741	− 0.0857
	Secondary	−0.2824	0.0443	−0.3705	− 0.2829	− 0.196
	Higher	−0.4105	0.0616	−0.5269	− 0.4119	− 0.2849
Mother’s occupation	Working_woman	0.025	0.0458	−0.0633	0.0269	0.1144
Father’s occupation	Laborer	0.1637	0.0703	0.025	0.1635	0.3027
	Farmer	−0.0581	0.0734	−0.2078	− 0.0583	0.0865
	Official	0.084	0.0698	−0.0547	0.0855	0.2174
	Businessman	0.0069	0.0712	−0.1376	0.0086	0.1499
Wealth index quantile	Second	−0.1842	0.0441	−0.2677	−0.1838	− 0.0974
	Middle	−0.3471	0.0533	−0.4508	−0.3467	− 0.2429
	Fourth	−0.4489	0.0636	−0.5748	−0.45	− 0.3224
	Highest	−0.8336	0.0837	−1.0036	−0.8334	−0.6657
Total number of under-five children in household		0.0138	0.0137	−0.0128	0.0135	0.0402
Total children ever born to a woman		0.0392	0.0088	0.022	0.0391	0.0566
Sanitation	Improved	−0.0894	0.0362	−0.1579	− 0.0899	−0.0181
Sources of drinking water	Improved	−0.0261	0.0657	−0.1507	−0.0274	0.1076
Access to media	Yes	−0.024	0.0329	−0.0886	−0.0234	0.0418
Region	Central Punjab	−0.3928	0.0643	−0.5236	−0.3912	− 0.27
	Northern Punjab	−0.0868	0.0311	−0.1458	−0.0875	− 0.024

**Table 3:** Smooth terms variances

Smooth Terms	Mean	Sd	2.50%	50%	97.50%	Min	Max
Age of children	0.0209	0.0617	0.0005	0.0054	0.1284	0.0001	2.2943
Age of mother when children born	0.0028	0.0593	0.0003	0.001	0.0062	0.0001	2.6521

According to summary of results (Table 2), going from rural to urban areas decreases the log odds of underweight by 0.13.

Underweight status of Children under-five in Punjab is negatively associated with education level of father and mother of children. The log odds of underweight decreased by 0.09, 0.17, 0.28 and 0.41 for children whose fathers attain primary, middle, secondary and higher level of education respectively w.r.t. those having no education at all. The log odds of underweight decreased by 0.08, 0.19, 0.25 and 0.59 for children whose mothers got primary, middle, secondary and higher education respectively, compared to those having no education (Table 2).

The risk of underweight is significantly affected by father’s occupation. Here, unemployed category is taken as a benchmark. The log odds of underweight are increased by 0.08 and 0.16 for the children whose fathers were officials and laborer respectively; in contrast, they decreased by 0.06 for child whose fathers were farmers and remained almost unchanged for children whose fathers owned any business entity (small or large) (Table 2).

Underweight status of Children under-five in Punjab is negatively correlated with household wealth index quintile. Going from the lowest wealth index quartile to second, middle, fourth and highest wealth index quantiles, decreases the log odds of underweight by 0.18, 0.35, 0.45 and 0.83 respectively (Table 2).

Total number of children ever born to a woman is positively associated with the risk of underweight. Addition of every single child into the total number of children ever born to a woman increases the log odds of underweight by 0.04 (Table 2).

The household’s access to improved sanitation facility decreases the log odds of underweight by 0.09 compared to unimproved sanitation facilities (Table 2).

**Fig. 2:**
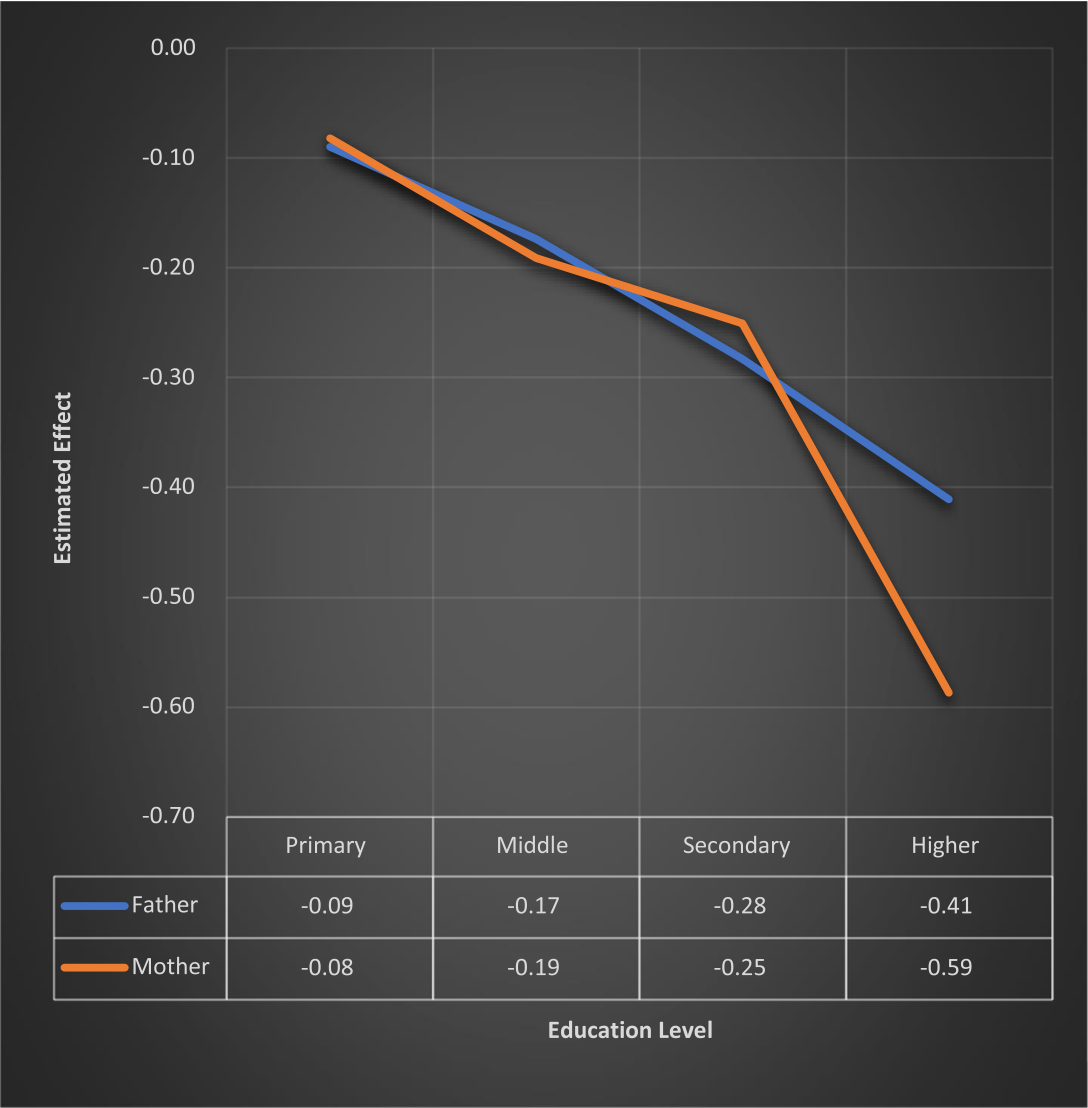
Comparison of Effect of Education Level of Mother” and Father” of Children on Underweight. A line chart developed through Microsoft excel to see the effect of education level of mother and father on underweight status of under-five children in Punjab province of Pakistan. Estimated effect is corresponding value of regression coefficient for each level of education

Underweight status of children is inversely related to the age of their mothers. The risk of underweight found highest in the children whose mother’s age was less than twenty years at time of their birth, and it decline down gradually as the age of mother increase. The spread of underweight is almost same for mother’s age group of 20–42 years (when children born), relatively higher beyond this age group, and highest if mother age is grater then 45 years (Fig. 2).

**Fig. 3:**
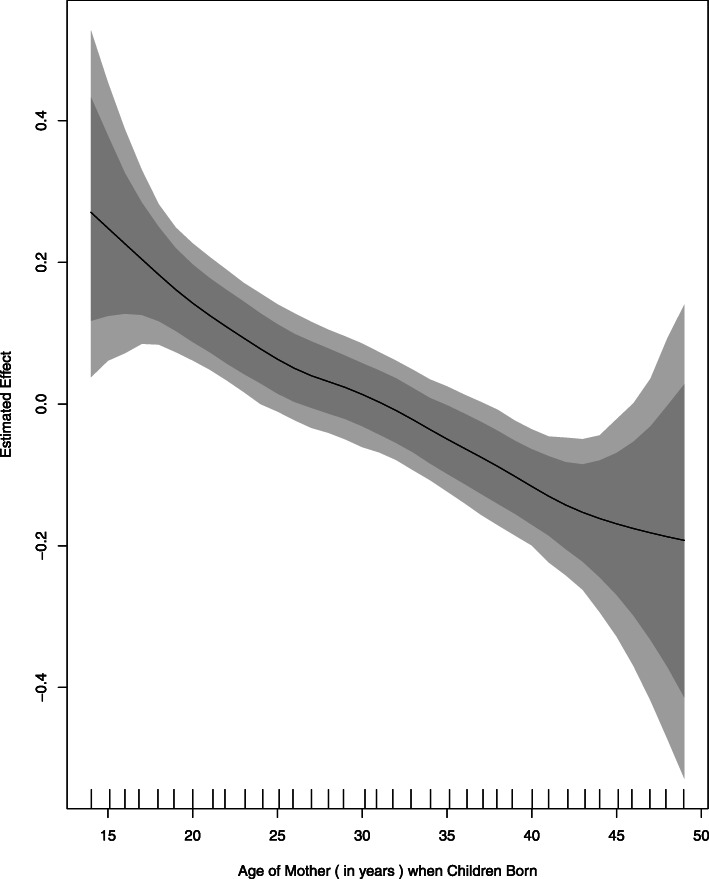
Effect of Mother Age when Children Born (In Years)” on Underweight. A Built-in function of BayesX package in R language (based on smooth term “sx (MACB)”, provided in Table 3), wherein Estimated Effect of Covariate “Mother Age when Children Born” i.e. “f (MACB)“ is plotted against corresponding values of covariates

Underweight status of children is directly but non-linearly correlated with the age of children (in months) as shown in Fig. 3 and Table 3. It shows that underweight status of children is normal from birth up to about 12 months of age, then it gradually inclines and got severe after 18 months of age. This pattern continues until the age of 20 months and turn out to be almost static up to the age of about 42 months, afterward falls sharply. The prevalence of underweight is revealed to be more pronounced in age group of 18–32 months (Fig. 3).

Exposure of child’s mother to mass media reduces the log odds of underweight by 0.02 in comparison with no access to media at all (Table 2).

Refer to Table 2, the prevalence of underweight is more noticeable in the Southern Punjab. Going from Southern Punjab to Central and Northern Punjab decreases the log odds of underweight by 0.09 and 0.39.

The underweight status of children is slightly but not significantly affected by mother’s occupation, source of drinking water and total number of under-five children in household (Table 2).

## Discussion

This study is focused on various socioeconomic and demographic aspects of underweight prevalence among under-five children in Punjab province of Pakistan and may facilitate the concerned administration and policy makers in formulation of apt polices to address it.

However, it exclude others factors related to health of children and mothers such as birth interval, health status of mother during pregnancy, place of delivery, weight of a child at birth, exclusive breast feeding, vaccination status of children, child morbidity status (fever, measles, diarrhea and ARI), type of food he/she received, mother’s BMI, maternal and eternal care and use of extra food during pregnancy etc. In addition, there are three commonly used measures of child nutritional status: Stunting (height-for-age), Wasting (weight-for-height) and Underweight (weight-for-age). For the sake of conciseness only the last mentioned has been used, in this study.

Since, quality, availability and accessibility of hygienic food, health care and other facilities (like water and sanitation, exposure to media etc.) are considerably lower in rural Punjab, therefore, the children from rural areas of Punjab found more like to be underweight than their urban counterparts. This finding is consistent with findings obtained by other authors [12, 14, 17].

**Fig. 4:**
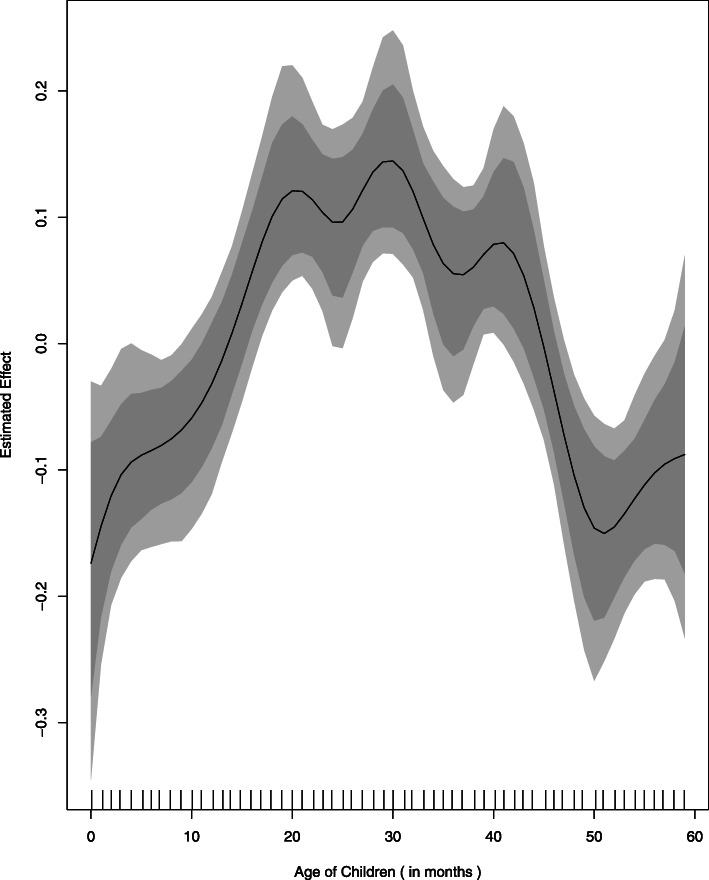
Effect of Children Age (In Months)” on Underweight.A Built-in function of BayesX package in R language (based on smooth term “sx(CAGE)”, provided in Table 3), wherein Estimated Effect of Covariate “Age of children in months” i.e. “f (CAGE)“is plotted against corresponding values of covariates

Our finding discloses a strong positive association between underweight and education level of father and mother of children. It is because, the educated parents usually have more knowledge about diet and health of children. In addition, education may change their traditional beliefs about diseases and taking care of their children. It also found consistent with several other similar studies [5, 6, 10, 12, 14–18]. Furthermore, the risk of underweight is 18% lower in children whose mother possesses higher education than fathers with same educational qualification (Fig. 4). It might because, the mothers usually spend more time with children compared with their fathers.

The risk of underweight found more severe in children belongs to families with lowest wealth index quintile. Because, it is impossible for parents with a very small income to maintain the increasing need of food as well as health expenditures of children, which results undernourishment. On the other hand, wealthier parents are more likely to afford hygienic food, healthier living conditions and better health-care that improves the child nutrition and overall health. Many other studies on child nutrition illustrate the similar results [5, 10, 12–16].

Our study has found positive relationship between the number of children ever born to a woman and underweight prevalence. Same findings are revealed in a study carried out by Islam et al. (2013) [12]. Generally, the number of children ever born to a woman (fertility) have an inverse effect on child nutrition and health, which results the child to be underweight. It is because, families with more children, experience more economic strain for food consumption; hence, the children from these families are more likely to suffer from malnutrition and underweight. In addition, up to 40% of people in Punjab fall in 1St and 2nd wealth index quintile, i.e. low-income quintiles (Table 4). Whereas, this percentage goes up to 45% for families with one or more children in household under-five year of age. Therefore, it is impossible, for such people to maintain their livelihood and fulfill their basic needs. Hence, addition of every newborn baby in family may further reduce the distribution of income on health and food of children, consequently the risk of underweight increase.

**Table 4:** Wealth index quintile

Wealth Index Quintile						
**Quintiles**	**Overall**			**Families of children under-five (at least one)**		
	**Frequency**	**Percentage**	**Cumulative %**	**Frequency**	**percentage**	**Cumulative %**
Lowest	48,469	19.7	19.7	5685	23.4	23.4
Second	49,543	20.1	39.8	5133	21.1	44.5
Middle	51,590	20.9	60.7	4988	20.5	65.1
Fourth	50,823	20.6	81.3	4833	19.9	85
Highest	46,076	18.7	100	3655	15	100
**Total**	**246,501**	**100**	**–**	**24,294**	**100**	**–**

The underweight status of children found negatively associated with improvement in sanitation facilities. It is because, the improved sanitation reduces the risk of diseases; consequently, the risk of underweight is decreased. The source of drinking water revealed similar but comparatively lesser impact on underweight status of children. It might because, the 94% inhabitants of Punjab use improved source of drinking water while comparatively lesser proportion of population (66%) use improved sanitation facility [1]. In addition, the ‟improved source” indicator is based on the categorization of water supplies by type of facility and does not consider the direct measurements of water quality, i.e. the water from improved source is not necessarily be free from contaminants [23].

The underweight status of children is negatively associated with mother’s access to media (Table 2). It could be because, the exposure to media may lead to educate the mothers about health and food of children as well as more effective allocation of income to devote on their health and nutrition. These results are consistent with findings obtained by other authors [12, 17].

Refer to Fig. 4, the underweight status of children is inversely related to age of mother when child born. The surprising result for the underweight status of children whose mother age is greater than forty years is probably due to smaller frequency of observations with very high dispersion among them. Mothers of only 2.8% of children had the age 40 years or more when they born as well as the variation among the risk of underweight relatively higher for this age group.

The reasons for nonlinear association of underweight status of children with their age are that: usually children born with approximately normal anthropometric status; but afterwards, the health status of the most children became worse due to different socio-economic and biological factors and until it improved and become stable at a low level. The highest risk of underweight during the age group of 18–32 months is might because, during the aforesaid age group, kids are usually weaned from breast as well as their mothers do lose their ability to produce enough milk to fulfill the nutritional requirement of growing children. These results are consistent with findings obtained by other authors [10, 11, 14].

The causes for such a high rate of underweight prevalence in Southern Punjab Southern Punjab also called Seraiki belt (consisting of 11 districts) could be:

Firstly, Southern Punjab is more deprived region of province, 41% of people residing in this region have lowest wealth index quintile (Table 5).

**Table 5:** Wealth index quintile, by Region

Region	Wealth Index Quintile (Row %)				
	Lowest	Second	Middle	Fourth	Highest
Southern	40.9	21.7	16.6	12.6	8.2
Central	15.3	21.6	22.8	22.8	17.4
Northern	6.1	14.9	21.0	30.3	27.7

Secondly, 72% of people living in Southern Punjab belongs to rural areas with poor health, living and educational facilities which is highest in entire province (Table 6).

**Table 6:** Urban-Rural distribution of sampled population, by Region

Region	Locality (Row %)	
	Rural	Urban
Southern	72.3	27.7
Central	63.5	36.5
Northern	57.7	42.3

Thirdly, the statistics of parent’s education of under-five children is worse in Southern Punjab. In this region, 64% of mothers and 41% of fathers of under five children are illiterate (Table 7). This percentage is highest in entire province (Table 7).

**Table 7:** Education Level of Mother and Father, by Region

Region	Mother’s Education (Row %)					Father’s Education (Row %)				
	None	Primary	Middle	Secondary	Higher	None	Primary	Middle	Secondary	Higher
Southern	64.1	14.7	6.8	7.4	7.0	41.3	19.0	13.3	15.1	11.2
Central	42.7	20.7	10.9	13.7	12.0	23.5	18.8	17.9	24.6	15.3
Northern	28.0	19.8	13.2	18.9	20.1	10.4	14.0	22.4	33.0	20.2

Fourthly, 45% of the inhabitant of Southern Punjab have no access to improved sanitation which is also highest in entire province (Table 7).

Fifthly, about one-half of resident of this region has no access to mass media at all. It is not only highest compared with rest of Punjab but horrible to see in this era (Table 8).

**Table 8:** Access to Mass Media and Sanitation, by Region

Region	Sanitation (Row %)		Access to Media (Row %)	
	Unimproved	Improved	No	Yes
Southern	44.5	55.5	50.0	50.0
Central	29.8	70.2	29.2	70.8
Northern	19.3	80.7	20.4	79.6

In addition, Southern Punjab is considered as most deprived and neglected part of Punjab. Since 1970, South Punjab has not been receiving its due share from provincial resources. Development schemes for said region were diverted to Central and Northern parts of province. According to recent data, poverty and unemployment are relatively higher in this region [1, 3, 24–27]. Furthermore, unlike rest of Punjab province, People in Southern part of province, which are under the sway of powerful landed gentry and tribal sardars (Tribal chiefs), suffer from lack of opportunity to improve their livelihood.

Therefore, to-a-days, for fair distribution of resources and administrative reasons, the demand for creation of new autonomous province in south Punjab, i.e. Seraiki Province (Seraiki is a native language of this region), is under national debate and focus.

## Conclusion

The results of this study reveal that the family’s wealth index quintile, mother education level, father education level, geographical region, locality and total number of children ever born to a woman (fertility) are significantly associated with underweight status of children under-five in Punjab. The results from this study are found to be consistent with other studies [6, 8, 11, 13].

In order to achieve the sustainable development goal Punjab needs to scale up target-oriented programs such as poverty reduction income-generating interventions and improvement of education and health facilities for poor population and deprived parts of province especially Southern Punjab.

Recommendations for researchers:

There are three commonly used measures of child nutritional status: Stunting, Wasting and Underweight. Only the last-mentioned measure has been used in this study. Hence, Stunting and wasting may also be used for said purpose. In addition, this study is specific to Punjab province; same may be don for other provinces/parts of Pakistan.

## Supplementary Information

**Additional file 1.** Supplementary information 1 [27–33]

**Additional file 2.** Supplementary information 2 [34–38]

**Additional file 3 **: **Figure B1** ACF for step = 1.Auto correlation function plot at step = 1 created through R language

**Additional file 4 **: **Figure B2** ACF for step = 10. Auto correlation function plot at step = 10 created through R language

## Abbreviations

ARI: Acute Respiratory Illness
BMI: Body Mass Index
HH: Household
MDGS: Millennium Development Goals
MICS: Multiple Indicator Cluster Survey
NNS: National Nutrition Survey
UN: United Nations
UNICEF: United Nation Children’s Emergency Fund
WHO: World Health Organization
