# Supplementary material for: Socioeconomic and demographic factors determining the underweight prevalence among children under-five in Punjab
Source: BMC Public Health. 2020 Nov 30;20:1817. doi: 10.1186/s12889-020-09675-5 (PMC7708259; doi:10.1186/s12889-020-09675-5)
Supplement: Supplementary file 1 — Additional file 1. Supplementary information 1 [27–33] [file 12889_2020_9675_MOESM1_ESM.pdf]

<u>**APPENDIX -A**</u>

Table A1: Description of covariates

Covariate	Description	Type	Categories
Locality	Place of residence of children	Categorical	1. Rural<br>2. Urban
Gender	Gender of children	Categorical	1. Female<br>2. Male
melevel	Mother's education Level of	Categorical	1. None<br>2. Primary<br>3. Middle<br>4. Secondary<br>5. Higher
felevel	Father's education Level of	Categorical	1. None<br>2. Primary<br>3. Middle<br>4. Secondary<br>5. Higher
f_ocp	Father's occupation	Categorical	1. Unemployed<br>2. laborer<br>3. Farmer<br>4. official<br>5. Businessman
m_ocp	Mother's occupation	Categorical	1. Housewife<br>2. Working Woman
DW	Sources of drinking water	Categorical	1. Unimproved<br>2. Improved
CM10	Total children ever born to a woman	Discrete	None
HH14	Total number of under-five children in household	Discrete	None
MACB	Age of mother in years when children born	Continuous	None
CAGE	Age of children in months	Continuous	None
AMedia	Access to mass media	Categorical	1. Yes<br>2. No
Sanitation	Type of sanitation	Categorical	1. Unimproved<br>2. Improved
Windex5	Wealth index quintile of family	Categorical	1. Lowest<br>2. second<br>3. Middle<br>4. fourth<br>5. Highest
Region	Geographical region	Categorical	1. South Punjab<br>2. Central Punjab<br>3. Northern Punjab

EDUCATION LEVEL

‟Education level” means the highest level of education attained by the father or mother of child. None refer to no education at all, ‟Primary” refer to 1 to 5 years of education, ‟Middle” 6 to 8 years of education, ‟Secondary” 9 to 10 years of education while ‟Higher” refer to 11 years and above.

OCCUPATION

Occupation means major income source of father or mother of children. The occupation of father and mother of children are taken separately.

Persons in employment are defined as “all those of working age who, during last month (from date of interview), were engaged in any activity to produce goods or provide services for pay or profit”. Here, Employment linked to what a person does (economic activity), not what he received. Therefore, a Retired person receiving pension but not involved in any other economic activity was also considered as an unemployed. “Farmer” refers to a person who was involved in agriculture related activities, livestock, forestry or forming. “Official” include; Government / Semi Government Employee and Private Employee receiving monthly salaries. “Businessman “relates to a person that works or look after the business entities owned by him, at any level (small or large). “Laborers” refer to daily wage workers.

IMPROVED SOURCES OF DRINKING WATER

- Piped water
- Borehole
- Protected well
- Tube well
- Rainwater collection
- Protected spring

UNIMPROVED SOURCES OF DRINKING WATER

- Bottled water
- Rivers or ponds
- Tanker truck water
- Unprotected spring
- Unprotected

Bottled water is considered as an improved water source only if improved water source is being used in household for cooking and hand washing. Otherwise it is considered as unimproved because of limitations in the potential quantity, not quality, of the water.

IMPROVED SANITATION FACILITIES

- Connection to a septic system or public sewer
- Simple pit latrine
- Pour-flush latrine
- Ventilated pit latrine

UNIMPROVED SANITATION FACILITIES

- Open pit latrine
- Public or shared latrine
- Bucket latrine

ACCESS TO MEDIA

Access to media is a composite index. It is constructed by three mass media variables, namely; whether the mother of children read newspaper /magazine, listen to radio or watch television. This is classified into two groups "Yes" and "No". 'Yes' means she has exposure to at least one of these media and 'No' mean no access to any of them at all.

WEALTH INDEX

Wealth index is also considered as a composite indicator of wealth. It constructed by utilizing the information of dwelling characteristics, ownership of consumer goods, water and sanitation, and other characteristics related to the wealth of household. Principal component analysis is performed to generate weights for each of the items used. An individual wealth score is assigned to each household in sample on basis of the assets it owned. All household are then ranked according to their wealth score in and finally divided into five quantiles from lowest to highest.

For the complete list of items/assets used in the calculation of this Wealth index, see MICS-2014 pp.15 [53]. For detailed discussion on the construction of the wealth index quintile see [28,29,30]

REGION

Unfortunately, there is no authorized scheme for regional classification of Punjab. Different authors have used different classification schemes, see [31,32,33,34].

Punjab province of Pakistan consists of thirty-six administrative districts. So, in this study, spatial aggregation the analysis is carried out on district level. In this regard, the entire province is divided into three geographical regions, viz. Central Punjab, Northern Punjab and Southern Punjab, based on the classification scheme suggested by Gazdar (1999) [30].

Table A2: Classification of Districts by Geographical Regions/ Spatial aggregation

Southern Punjab (District)	Central Punjab (District)		Northern Punjab (District)	
Bahawalpur	Faisalabad	Lahore	Rawalpindi	
Bahawalnagar	Jhang	Kasur	Attock	
R Y Khan	T.T.Singh	Nankana Sahib	Chakwal	
D.G.Khan	Chiniot	Sheikhupura	Jehlum	
Layyah	Gujranwala	Sahiwal	***Total Districts***	***36***
Muzaffargarh	Gujrat	Pakpatttan	***Southern Punjab***	***11***
Rajanpur	Hafizabad	Okara		
Multan	Mandi-Bahauddin	Sargodha	***Central Punjab***	***21***
Khanewal		Bhakkar		
Lodhran	Narowal	Khushab	***Northern Punjab***	***04***
Vehari	Sialkot	Mianwali		
