# Supplementary material for: Socioeconomic and demographic factors determining the underweight prevalence among children under-five in Punjab
Source: BMC Public Health. 2020 Nov 30;20:1817. doi: 10.1186/s12889-020-09675-5 (PMC7708259; doi:10.1186/s12889-020-09675-5)
Supplement: Supplementary file 2 — Additional file 2. Supplementary information 2 [34–38] [file 12889_2020_9675_MOESM2_ESM.pdf]

APPENDIX -B

STATISTICAL MODEL

$$logit[P(UWa) = 1] = \gamma_{\circ} + \gamma_1\, LocalityUrban + \gamma_2 melevelPrimary + \gamma_3\, melevelMiddle + \gamma_4\, melevelSecondary + \gamma_5\, melevelHigher + \gamma_6\, felevelPrimay + \gamma_7\, felevelMiddle + +\gamma_8\, felevelSecondary + \gamma_9\, felevelHigher + \gamma_{10}\, m\_\text{ocpWorkingWoman} + \gamma_{11}\, f\_ocpLaborer + \gamma_{12}\, f\_ocpFormer + \gamma_{13}\, f\_ocpOfficial + \gamma_{14}\, f\_ocpBusisnessnman + \gamma_{15}\, Windex5Second + \gamma_{16}\, Windex5Middle + \gamma_{17}\, Windex5Fourth + \gamma_{18}\, Windex5Highest + \gamma_{19}\, HH14 + \gamma_{20}\, CM10 + \gamma_{21}\, SanitationImproved + \gamma_{22}\, DW + \gamma_{23}\, AMediaYes + \gamma_{24}\, RegionCenteral + \gamma_{25}\, RegionNorthren + f_1(MACB) + f_2(CAGE)$$

Statistical analysis is performed on Statistical package R using BayesX and R2BayesX Libraries.

CALLING BAYESX FROM R

In R2BayesX library, the core model fitting function is called bayesx(). The arguments of bayesx() are like this:

bayesx (formula, family = "binomialprobit", data=data, method = "MCMC", iterations = 20000, burnin = 2000, step = 10)

In BayeX, estimation is carried out via three inferential concepts (briefly describe in next section). In above cited model fitting function, "Formula" refers to symbolic illustration of model like y~x. Arguments like iterations, burnin, step and family established the number of iterations of the MCMC simulation. The "burn-in period" mean the number of iterations which will be discard from the generated samples. The burn-in phase is normally required to achieve convergence of the Markov chain regarding its stationary (i.e. the posterior) distribution.

"Step" is the thinning parameter for MCMC simulation. It defines size for which samples should be stored. If, for example, step = 20, it implies that every 20th sampled parameter will be saved to estimate the characteristics of posterior distribution. The purpose of thinning is to grasp a significant reduction of disk storage and autocorrelations amongst sampled parameters.

For an effective fit, usually 12000 iterations are enough with non-significant autocorrelations of stored parameters, at least in the model building phase. However, it is worthwhile to take a higher number of iterations for the ultimate model that used for conclusions.
‟Family” refer to response distribution and link function. A massive range of distributions is available in BayesX as a family for distribution.

ESTIMATION OF STRUCTURE ADDITIVE REGRESSION MODELS WITH BAYESX

In ‟BayesX”, the estimation of regression coefficients is based on following three inferential concepts:

1. **Fully Bayesian Approach**

A fully Bayesian approach of Structure Additive Regression Models is obtained by allocating prior distributions for all unknown parameters. Usually, Multivariate Gaussian distribution is used as a joint prior distribution for the coefficient. Estimation is don through MCMC simulation methods. For detail, see Brezger et al. (2005) [35].

2. **Inference Via Mixed Model Representation**

Mixed model methodology is another approach to estimate Structure Additive Regression Models. The general concept is to make use of the close link between penalty concept and respective random effects distributions. In this approach, the smoothing variances of the priors are transformed to variance components. Whereas, regression coefficients form the basis for the determination of smoothing parameters. From a Bayesian viewpoint, this yields empirical Bayes/posterior approach estimates for the Structure Additive Regression Models. However, from frequents perspective estimates can also be interpreted as a penalized likelihood estimate [25].

3. **Penalized Likelihood Including Variable Selection**

BayesX offers a penalized likelihood approach as another alternative for estimation of Structure Additive Regression models. In this approach, model selection and parameters estimation are carried out simultaneously through a powerful, built-in variable and model selection algorithm.
The algorithm of penalized likelihood can decide: whether a specific variable move in the model, whether a continuous variable come in in the model linearly or nonlinearly, and whether a spatial effect introduce in the model. It is also able to select the degree of smoothness of nonlinear covariate or spatial effects.

CONVERGENCE DIAGNOSTICS

There are various ways to detect the convergence of MCCM. In this study, we use Geweke test and auto correlation function (ACF) of sampled parameters. "Coda" package of R language is used for this regard. For detailed discussion on different convergence techniques, see [8,36,37,38].

The Geweke diagnostic (proposed by Geweke (1992)) consider two non-intersecting parts (normally the first 10% and last 50% portions) of the markov chain and use a test for equality of the means to see whether the both parts of the chain belongs to the same distribution (null hypothesis). The standard Z-score is test statistic for Geweke test with standard error adjusted for autocorrelation. If the mean of the initial 10% part of chain is not significantly different from the last 50%, it implies that the target distribution converged anywhere in the first 10% of the chain, else diverge.

Initially we start with only 1000 iterations. The values of Geweke statics along with corresponding p values are shown in table-B1. It shows that for 1000, the markov chains for 3 coefficients are not converging toward stationary distribution. So, we use 1000 iterations, run the model and apply Geweke test. For 1000 iterations, the markov chains pertaining to two coefficients, viz.: Sanitation (improved) Region(northern) are still not converging toward stationary distribution. Since, total convergence has not achieved up to 10000 iterations; so, it is obligatory to use some higher number of iterations. For 15000 iterations, markov chains for all coefficients are converging toward stationary distribution. Result is same for 20000 iterations.

Summarizing the results, we reached on conclusion that for adequate fit, 20000 iterations are enough for this model. Since the values of the geweke statistic for all coefficients are not statistically insignificant, so burn-in period is not required. Hence, we set burn-in = 0.

In order to detect the autocorrelations amongst sampled parameter, we take step= 1 and 10 respectively. For step = 1 the autocorrelations amongst all sampled parameters are seemed extremely significant (figure B1). In contrast, for step = 10, autocorrelations are relatively much lower. In addition, apart from few initial spikes of ACF, almost all of them are not significant up to lag = 50 (figure B2). Therefore, we used step=10 for our final model.

Appendix B: Geweke Statistic

Coefficients		Iterations							
		1000		10000		15000		20000	
		Z-score	P-value	Z-score	P-value	Z-Sore	P-Value	Z-Sore	P-value
Intercept		2.9796	**0.0029**	-0.0294	0.9766	0.2328	0.8159	0.1278	0.8983
Locality	Urban	-0.0217	0.9827	-0.9244	0.3553	-0.9454	0.3445	-1.0903	0.2756
Mother's education level	Primary	-0.6502	0.5156	-0.0235	0.9812	-1.3364	0.1814	-1.2106	0.2261
	Middle	-1.0325	0.3018	0.0568	0.9547	-0.4347	0.6638	-0.2301	0.8181
	Secondary	-0.2848	0.7758	-0.8359	0.4032	-1.595	0.1107	-1.9383	0.0526
	Higher	0.5247	0.5998	0.7801	0.4353	0.0119	0.9905	-0.3506	0.7259
Father's education level	Primary	-0.7713	0.4405	-1.1807	0.2377	0.4159	0.6775	0.4047	0.6857
	Middle	-0.2186	0.827	-0.0491	0.9609	0.7504	0.453	0.09	0.9283
	Secondary	-0.3382	0.7352	0.4181	0.6758	1.7564	0.079	1.1101	0.2669
	Higher	-0.3085	0.7577	-0.4723	0.6368	0.8208	0.4118	0.8037	0.4216
Mother's occupation	Working_ woman	0.085	0.9323	0.9636	0.3353	1.3687	0.1711	1.3437	0.1791
Father's occupation	Laborer	-0.1937	0.8464	0.5906	0.5548	0.6823	0.4951	1.0201	0.3077
	Farmer	-0.0454	0.9638	0.5641	0.5727	0.7391	0.4599	0.9606	0.3367
	Official	-0.3075	0.7584	0.5844	0.5589	0.6737	0.5005	0.9596	0.3373
	Businessman	-0.292	0.7703	0.6867	0.4923	0.7183	0.4726	1.1609	0.2457
Wealth index quantile	Second	-0.4726	0.6365	1.6811	0.0928	1.3623	0.1731	1.6917	0.0907
	Middle	-0.1108	0.9118	1.8569	0.0633	1.3189	0.1872	1.5326	0.1254
	Fourth	-0.0327	0.9739	1.6061	0.1083	1.0827	0.2789	1.3899	0.1646
	Highest	-0.1779	0.8588	1.3742	0.1694	0.767	0.4431	0.8133	0.416
Total number of under-five children in household		-1.1282	0.2592	-1.951	0.0511	-1.931	0.0535	-1.4472	0.1478
Total children ever born to a woman		-2.1557	**0.0311**	0.6287	0.5296	-0.5444	0.5862	-1.0871	0.277
Sanitation	Improved	0.1362	0.8917	-2.4598	**0.0139**	-1.7873	0.0739	-1.6579	0.0973
Sources of drinking water	Improved	-3.6416	**0.0003**	-0.509	0.6107	-1.1121	0.2661	-0.8136	0.4159
Access to media	Yes	-1.1427	0.2532	-0.9028	0.3666	-0.22	0.8259	-0.985	0.3246
