# Supplementary figures and images for: Socioeconomic and demographic factors determining the underweight prevalence among children under-five in Punjab
Source: BMC Public Health. 2020 Nov 30;20:1817. doi: 10.1186/s12889-020-09675-5 (PMC7708259; doi:10.1186/s12889-020-09675-5)

# Maximum autocorrelation of sampled parameters

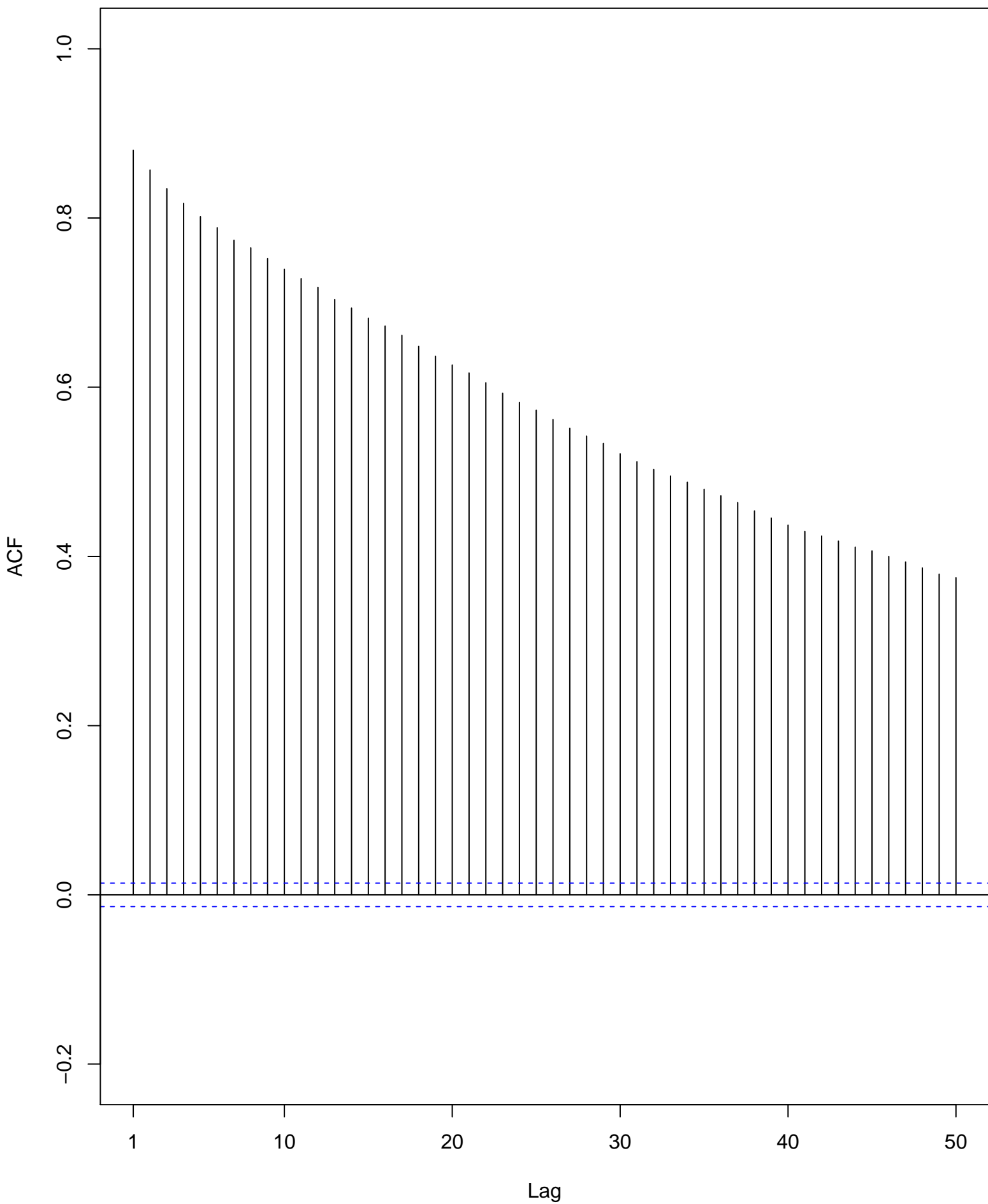

Supplement: Supplementary file 3 — Additional file 3 : Figure B1 ACF for step = 1.Auto correlation function plot at step = 1 created through R language [file 12889_2020_9675_MOESM3_ESM.pdf]

# Maximum autocorrelation of sampled parameters

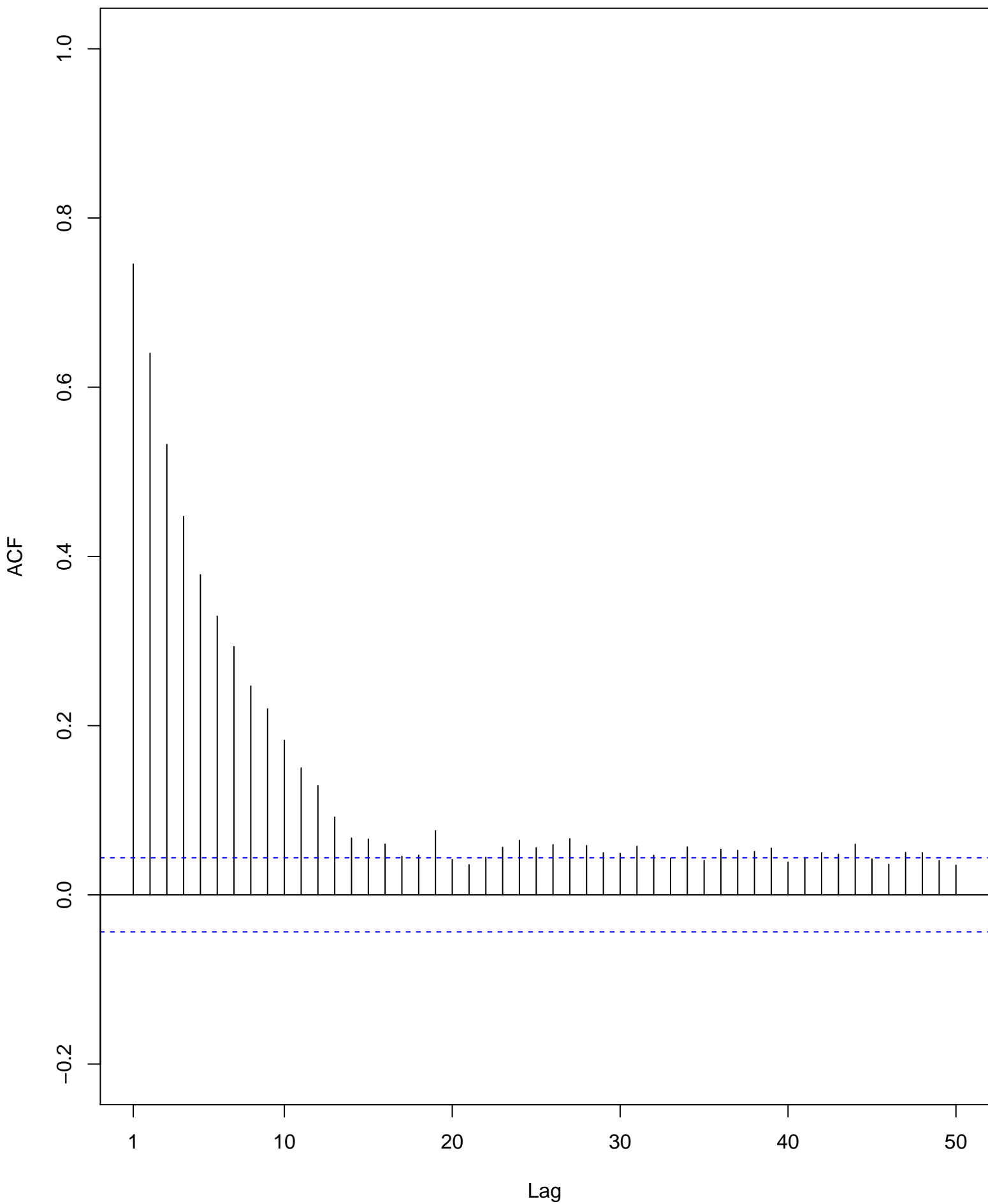

Supplement: Supplementary file 4 — Additional file 4 : Figure B2 ACF for step = 10. Auto correlation function plot at step = 10 created through R language [file 12889_2020_9675_MOESM4_ESM.pdf]
